# Application of matrix-assisted laser desorption ionization time-of-flight mass spectrometry in the detection of vancomycin-resistant and-susceptible *Enterococcus faecium*

**DOI:** 10.3389/fmicb.2025.1603986

**Published:** 2025-06-06

**Authors:** Wei He, Xintong Lin, Xueqin Chen, Liangming Zeng, Xuemin Guo

**Affiliations:** ^1^Institute of Basic Medical Sciences, Meizhou People’s Hospital, Meizhou, Guangdong, China; ^2^Guangdong Engineering Technological Research Center for Clinical Molecular Diagnosis and Antibody Drugs, Meizhou, Guangdong, China; ^3^State Key Laboratory of Neurology and Oncology Drug Development, Nanjing, Jiangsu, China; ^4^Affiliated Meizhou Hospital of Shantou University Medical College, Meizhou, Guangdong, China; ^5^Clinical Laboratory Center, Meizhou People's Hospital, Meizhou, Guangdong, China; ^6^Guangdong Provincial Clinical Research Center for Laboratory Medicine, Guangzhou, Guangdong, China

**Keywords:** MALDI-TOF MS, vancomycin sensitive, vancomycin resistance, *Enterococcus faecium*, characteristic peaks

## Abstract

**Introduction:**

In recent years, the escalating prevalence of Vancomycin-resistant *Enterococcus faecium* (VREfm) has emerged as a formidable challenge to global healthcare systems. While Matrix-assisted laser desorption/ionization time-of-flight mass spectrometry (MALDI-TOF MS) has become an indispensable tool for bacterial identification, its potential for rapid discrimination between Vancomycin-susceptible *Enterococcus faecium* (VSEfm) and VREfm through characteristic peak analysis remains an area of active investigation.

**Methods:**

In this study, we conducted literature search through databases to summarize the distribution of regionally prevalent VSEfm/ VREfm characteristic peaks, and further collected mass spectrometry data from Meizhou People’s Hospital (Guangdong, China) from 2021 to 2024 to explore stable characteristic peaks for both VSEfm and VREfm.

**Results:**

Through MALDI-TOF MS analysis, we identified stable characteristic peaks for both VSEfm (m/z 3299.95 ± 3.99 and m/z 6605.13 ± 7.28) and VREfm (m/z 3313.01 ± 2.76 and m/z 6631.03 ± 4.38) in the Meizhou region, with their discriminative efficacy validated by ROC curve analysis.

**Discussion:**

Our findings not only demonstrate the regional specificity of these characteristic peaks but also establish a robust methodological framework for rapid VSEfm/VREfm differentiation. This advancement holds significant promise for guiding clinical decision-making and controlling VREfm dissemination. Nevertheless, we acknowledge the necessity for ongoing technological refinement to enhance the accuracy and broader applicability of this approach in diverse clinical settings.

## 1 Introduction

*Enterococcus faecium* (*E. faecium*), a significant opportunistic pathogen in hospital-acquired infections, poses a serious threat to immunocompromised patients by causing diseases such as urinary tract infections, bloodstream infections, endocarditis, and intra-abdominal infections, significantly increasing patient morbidity and mortality (Wei et al., 2024; Getahun Strobel et al., 2024). In recent years, the global spread of vancomycin-resistant *E. faecium* (VREfm) has become increasingly severe, with its resistance posing a major challenge to clinical treatment. In light of this, the World Health Organization classified VREfm as a high-priority pathogen in 2017, highlighting its substantial threat to global public health security [Zhou et al., 2020; Segawa et al., 2024; Shen et al., 2024; World Health Organization (WHO), 2017]. In clinical practice, vancomycin remains the first-line antibiotic for treating *E. faecium* infections, while VREfm infections require alternative therapeutic options, such as linezolid and tigecycline. Therefore, establishing rapid and accurate methods to distinguish between vancomycin-susceptible *E. faecium* (VSEfm) and VREfm is crucial for guiding appropriate clinical antibiotic use and ensuring timely control of disease progression.

Currently, clinical detection of bacterial resistance primarily relies on conventional antimicrobial susceptibility testing (AST). However, this method is time-consuming and lacks immediacy. Matrix-assisted laser desorption/ionization time-of-flight mass spectrometry (MALDI-TOF MS) offers a more efficient solution. By analyzing protein fingerprint spectra, this technology not only enables rapid and accurate bacterial identification but also shows considerable promise for antibiotic resistance detection and epidemiological strain typing (Oviaño and Bou, 2018; Florio et al., 2018). Numerous studies have confirmed the successful application of this technology in analyzing drug resistance in various clinically important pathogens. Examples include detecting carbapenem resistance in *Klebsiella pneumoniae* (Centonze et al., 2018; Huang et al., 2022), identifying methicillin resistance in *Staphylococcus aureus* (Schuster et al., 2018; Kim et al., 2019), and assessing *β*-lactam resistance in pathogenic *Escherichia coli* (Hart et al., 2015; López-Cortés et al., 2024). Molecular assays, including but not limited to PCR or multiplex PCR in combination with gel electrophoresis, real-time fluorescence quantitative PCR, and loop-mediated isothermal amplification (LAMP), are also developed and used in the identification of strain species and resistance mechanisms (Huang et al., 2019; Ji et al., 2024). In contrast to AST, the PCR-based methods are fast and specific for determining target resistance genes. However, in contrast to MALDI-TOF MS, the PCR-based methods are still time-consuming, labor-intensive, performance-complicated, and cost-expensive. MALDI-TOF MS is an indispensable complementation to molecular assays (Boattini et al., 2025).

The presence and absence of specific peaks on MS spectra are commonly used to predict antibiotic-resistant strains. Different from the characteristic ribosomal proteins’ peak used for genus and species identification, strain-specific or resistance-specific proteins’ peak is believed to be ideal markers for resistance determination. Preliminary explorations of characteristic peaks for VSEfm and VREfm have been conducted in some healthcare facilitites. However, the reported characteristi peaks showed diversity in the different isolates from different regions (Candela et al., 2022; Nakano et al., 2014; Griffin et al., 2012; Wang et al., 2014; Gao et al., 2024; Wu et al., 2020; Lu et al., 2017; Li, 2015; Wang et al., 2021; Wang et al., 2022). This gap hinders the general identification and application of conserved characteristic peaks. It is urgent and necessary to perform systematic analysis and external validation of the reported characteristic peaks used in MALDI-TOF MS to distinguish VSEfm from VREfm.

In this study, we aim to develop and validate more stable characteristic peaks for both VSEfm and VREfm prediction. By employing bibliometric analysis, we comprehensively reviewed all the characteristic peaks reported for VSEfm or VREfm identification and explored whether the characteristic peaks were common or region-related. Next, by combining the consecutively collected MALDI-TOF MS data with the respective laboratory-confirmed antibiotic resistance profile of *E. faecium* isolates from Meizhou People’s Hospital, we extracted the characteristic peaks for VSEfm or VREfm prediction. Finally, by using an independent validation set of *E. faecium* isolates, we assessed the reliability of the characteristic peaks for VSEfm and VREfm prediction. Our results provided a rapid MS-based method for distinguishing VSEfm and VREfm strains in the Meizhou region, which could help early diagnosis and precise treatment of the patients with VREfm-infected diseases.

## 2 Materials and methods

### 2.1 Strain collection

From January 2021 to December 2024, a total of 296 *E. faecium* strains with duplicates removed were isolated from Meizhou People’s Hospital, including 134 VSEfm strains and 162 VREfm strains. The 169 strains first isolated in a chronological order, comprising 73 VSEfm strains and 96 VREfm strains, were used as the training set; the subsequently isolated 127 strains, comprising 61 VSEfm strains and 66, were used as the validation set.

### 2.2 Species identification

Various types of patient samples were collected and streaked onto Columbia blood agar plates (Autobio, Zhengzhou, China), which were then incubated at 37°C in a CO_2_ incubator for 18 h. Fresh single colonies were selected and smeared onto an MSP 96 target polished steel plate (Bruker Daltonics, Germany). Subsequently, 1 μL of 70% formic acid was added to the target plate and allowed to air-dry at room temperature, followed by the addition of 1 μL of HCCA matrix solution. After the sample-matrix mixture dried at room temperature, mass spectra were acquired and analyzed using a MALDI-TOF MS spectrometer in positive ion mode (Microflex LT mass spectrometer, Bruker Daltonics, Germany) (Marí-Almirall et al., 2017; Schulthess et al., 2013; Almuzara et al., 2016). The instrument parameters were set as follows: ion source electrode 1 voltage at 20 kV, ion source electrode 2 voltage at 18.1 kV, ion source focusing lens voltage at 6 kV; nitrogen laser frequency at 60 Hz; mass range from 2,000 to 20,000 m/z, with a total of 240 laser shots per sample spot for measurement. External calibration of the spectra was performed using bacterial test standard (Bruker Daltonics, Germany) solution.

### 2.3 Drug susceptibility analysis of *E. faecium*

A single typical colony of *E. faecium* was selected and suspended to a 0.5 McFarland standard. Following the operational guidelines provided by bioMérieux for the GP67 susceptibility card, the minimum inhibitory concentration (MIC) was determined using the VITEK 2 Compact fully automated microbial analysis system, with *Enterococcus faecalis* ATCC 29212 as the quality control strain. According to the 2023 standards of the Clinical and Laboratory Standards Institute (CLSI), strains with a vancomycin MIC value ≥32 μg/mL were preliminarily identified as VREfm. To ensure the accuracy of the identification, further verification was performed using the disk diffusion method (Oxoid, Basingstoke, UK) and the E-test (Autobio, Zhengzhou, China) gradient diffusion method.

### 2.4 PCR detection of the genes conferring vancomycin resistance in *E. faecium*

A single colony of *E. faecium* was inoculated into Brain Heart Infusion broth (Oxoid, Basingstoke, UK) and cultured at 37°C with shaking (200 rpm) for 18 h. Genomic DNA was extracted using the Gram-positive Bacterium Genomic DNA Extraction Kit (Solarbio Sciences, Beijing, China) according to the manufacturer’s instruction. The *vanA*, *vanB*, *vanD* and *vanM* genes were amplified individually by PCR using the primers specific to *vanA* (with sequences 5′-CATGACGTATCGGTAAAATC-3′ and 5′-ACCGGGCAGAGTAT TGAC-3′) (Yan et al., 2016), *vanB* (with sequences 5′-AAGCTATG CAAGAAGCCATG-3′ and 5′-CCGACAATCAAATCATCCTC-3′) (Elsayed et al., 2001), *vanD* (with sequences 5′-TTTGTAAAGCCTGC CCGTTC-3′ and 5′-CCAAGTATCCGGTAAATCTTC-3′) (Yan et al., 2016), and *vanM* (with sequences 5′-CAGAGATTGCCAACAA CATTGA-3′ and 5′-TCGGGAATTGTTATACCTGCTG-3′) (Sun et al., 2019). The PCR reaction system contained 10 μL 2 × Gold Multiplex PCR Mix (Canvax Biotech, Jiangsu, China), 1 μL template DNA, 0.4 μL each of forward and reverse primers (10 μM), and 8.2 μL nuclease-free water. Thermal cycling condition was 95°C for 5 min, 30 cycles of 95°C for 30 s, 52°C for 30 s, and 72°C for 1 min; followed by 72°C for 7 min. Amplification products were analyzed by 1.5% agarose gel electrophoresis, and positive amplicons were purified and sequenced by Sangon Biotech (Shanghai, China).

### 2.5 Data collection and processing for MALDI-TOF MS

Based on the antimicrobial susceptibility results of *E. faecium*, mass spectrometry data for VREfm and VSEfm were collected. The MALDI Biotyper software was used to confirm strain scores, and strains with scores <2.0 were excluded (Algahawi et al., 2024). Using flexAnalysis 3.0 software, the baseline of the protein mass peaks for all strains was adjusted to the lowest level, and the curves were smoothed. The mass spectra of VSEfm and VREfm were divided into two groups, and differences in peaks within the range of 2000–20,000 m/z were visually observed from the fingerprint spectra to identify characteristic peaks. The mass spectral peak data within the 2000–20,000 m/z range for all strains were exported to Excel for statistical analysis.

### 2.6 Statistical analysis of characteristic peaks and evaluation of differentiation performance

The peak values in the regions corresponding to the characteristic peaks were collected, and the mean and standard deviation (SD) were calculated using SPSS software (v.26.0), with the peak m/z values expressed as mean ± SD. Receiver Operating Characteristic (ROC) curves were plotted for the characteristic peaks, and the Area Under the Curve (AUC) values were obtained. A 2×2 contingency table was used to calculate sensitivity, specificity, positive agreement rate, negative agreement rate, and overall agreement rate. Additionally, the 95% confidence intervals (95% CI) for each diagnostic performance metric were computed.

## 3 Results

### 3.1 Distribution of characteristic peak regions between VSEfm and VREfm

**Figure 1:**
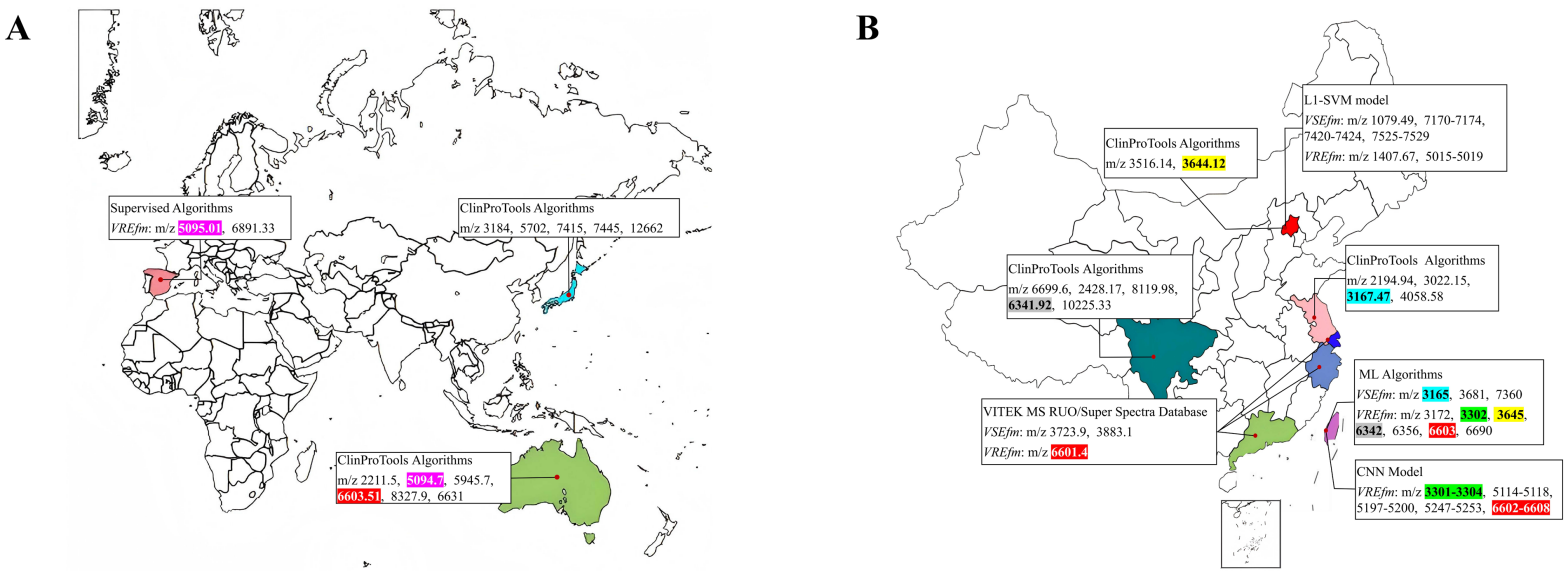
Regional distribution of characteristic peaks for VSEfm and VREfm (https://n.freemap.jp/). **(A)** Global analysis of characteristic peaks for VSEfm/VREfm across different regions. **(B)** Analysis of characteristic peaks for VSEfm/VREfm across different regions in China. Different peaks were highlighted in different colors while the common peaks were indicated in the same color: m/z 3,301–3,304 (green), m/z 3,165–3,168 (blue), m/z 3,644–3,645 (yellow), m/z 5,094–5,095 (purple), m/z 6,602–6,608 (red), m/z 6,341–6,342 (gray).

By searching databases, a comprehensive analysis was conducted on relevant literature published between 2012 and 2024 that reported the use of MALDI-TOF MS technology for rapidly distinguishing characteristic peaks of VSEfm and VREfm, totaling 10 studies (Candela et al., 2022; Nakano et al., 2014; Griffin et al., 2012; Wang et al., 2014; Gao et al., 2024; Wu et al., 2020; Lu et al., 2017; Li, 2015; Wang et al., 2021; Wang et al., 2022). The characteristic peak information of *E. faecium* primarily originated from Asia, Europe, and Oceania (Figure 1A). Notably, data from China covered multiple representative cities, including Beijing, Suzhou, Shanghai, Hangzhou, Chengdu, Guangzhou, and Taipei (Figure 1B). Various data analysis methods were employed across different regions, such as the ClinProTools algorithm, supervised algorithm, machine learning (ML) algorithms, L1-SVM support vector machine models, and CNN convolutional neural network models. These approaches successfully identified region-specific characteristic peak spectra while also revealing several conserved peaks, predominantly within the m/z ranges of 3,301–3,304, 3,165–3,168, 3,644–3,645, 5,094–5,095, 6,602–6,608, and 6,341–6,342 (highlighted in different colors in Figure 1). These findings suggest that the genetic evolution and spread of *E. faecium* strains exhibit distinct regional characteristics. Therefore, when applying characteristic peaks for resistance determination in clinical practice, it is essential to consider the significance of regional epidemiological features.

### 3.2 Selection and training of characteristic peaks for VSEfm/VREfm in the Meizhou area

**Figure 2:**
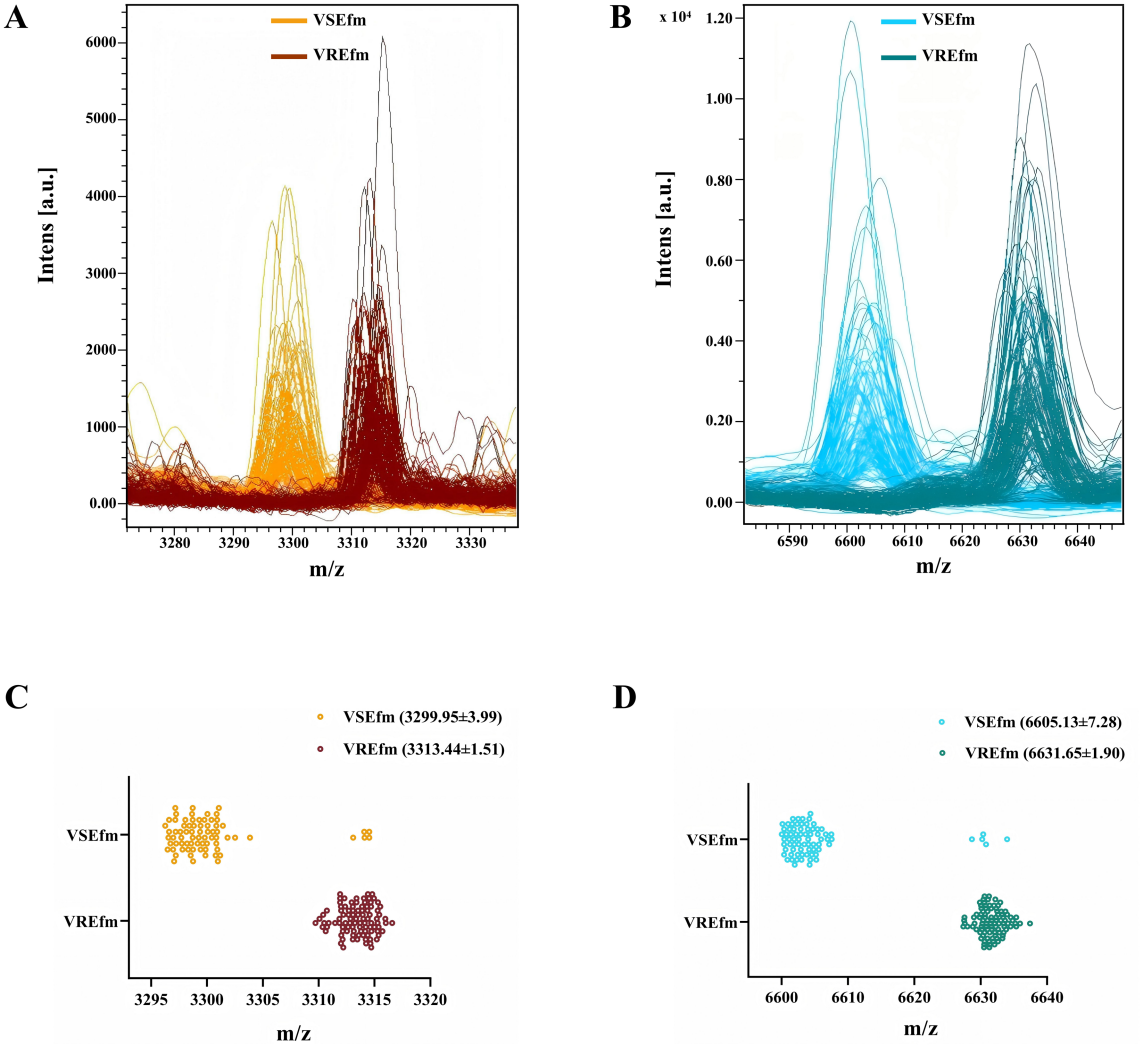
Rapid differentiation of VREfm and VSEfm strains in the Meizhou region based on the characteristic peaks. Mass spectra **(A)** and scatter plots **(C)** for the characteristic peaks at m/z 3299.95 ± 3.99 (VSEfm) and m/z 3313.44 ± 1.51 (VREfm), respectively. Mass spectra **(B)** and scatter plots **(D)** for the characteristic peaks at m/z 6605.13 ± 7.28 (VSEfm) and m/z 6631.65 ± 1.90 (VREfm), respectively. The values of all chacteristic peaks are presented with mean±SD. Color orange and cyan denote VSEfm; brown and dark cyan represent VREfm.

Mass spectrometry data of clinically isolated strains identified as *E. faecium* by the Bruker MALDI Biotyper mass spectrometer at Meizhou People’s Hospital from 2021 to 2024 were collected. Following validation by the MIC method for antimicrobial susceptibility testing, a total of 169 *E. faecium* strains were included as the training set, comprising 96 VREfm and 73 VSEfm strains. All VREfm strains were confirmed by PCR to carry the *vanA* genotype. Visualization analysis of the mass spectra of *E. faecium* strains revealed significant spatial shifts in two peaks: Peak ① was located at m/z 3299.95 ± 3.99 in VSEfm but shifted to m/z 3313.44 ± 1.51 in VREfm (Figures 2A,C); Peak ② was located at m/z 6605.13 ± 7.28 in VSEfm but shifted to m/z 6631.65 ± 1.90 in VREfm (Figures 2B,D).

**Figure 3:**
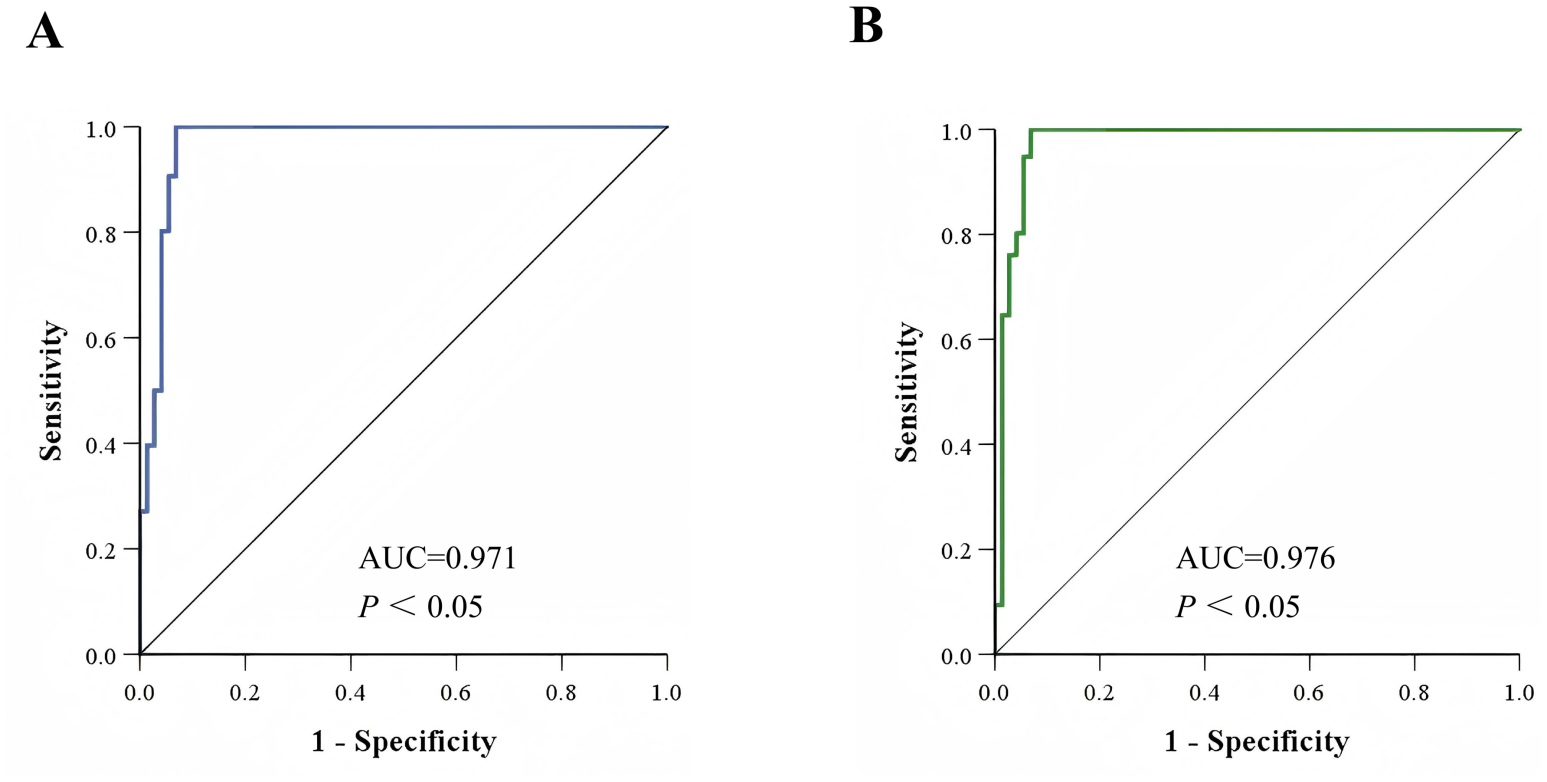
ROC curves of the characteristic peaks. **(A)** ROC curve of the characteristic peaks ranging from m/z 3299.95 ± 3.99 to m/z 3313.44 ± 1.51 used for identifying VSEfm and VREfm. **(B)** ROC curve of the characteristic peaks ranging from m/z 6605.13 ± 7.28 to m/z 6631.65 ± 1.90 used for differentiating VSEfm and VREfm.

To evaluate the differentiation performance of these characteristic peaks, ROC curves were generated using SPSS 26.0 software. The results showed that the AUC values for characteristic peaks at m/z 3299.95 ± 3.99 to m/z 3313.44 ± 1.51 and characteristic peaks at m/z 6605.13 ± 7.28 to m/z 6631.65 ± 1.90 were 0.971 (Figure 3A) and 0.976 (Figure 3B), respectively, with statistically significant differences (*p* < 0.05). A 2 × 2 contingency table comparing the identification results of the MIC method and the mass spectrometry method (Table 1) was constructed, and the diagnostic performance metrics for the two characteristic peaks were calculated as follows: sensitivity 100%, specificity 93.15% (95% CI: 87.36–98.95%), positive predictive value 95.05% (95% CI: 90.82–99.28%), negative predictive value 100%, and overall agreement rate 97.04% (95% CI: 94.49–99.60%) (Table 2). These results demonstrate that the identified characteristic peaks exhibit excellent capability in distinguishing between VSEfm and VREfm.

**Table 1:** 2×2 Contingency table for the training set based on identification results by MIC and mass spectrometry method.

Identification method		AST (MIC)		Total (Number of Strains)
		VREfm (Number of Strains)	VSEfm (Number of Strains)	
MALDI-TOF MS	VREfm (Number of Strains)	96	5	101
	VSEfm (Number of Strains)	0	68	68
Total (Number of Strains)		96	73	169

AST, antimicrobial susceptibility testing; MIC, minimum inhibitory concentration; MALDI-TOF MS, matrix-assisted laser desorption ionization time-of-flight mass spectrometry; VSEfm, vancomycin-susceptible *Enterococcus faecium*; VREfm, vancomycin-resistant *Enterococcus faecium*.

**Table 2:** Identification efficacy of VREfm and VSEfm characteristic peaks in the training set and the validation set.

Diagnostic performance metric		Sensitivity [95% CI]	Specificity [95% CI]	Positive predictive value [95% CI]	Negative predictive value [95% CI]	Accuracy [95% CI]
Training Set	VSEfm	93.15% [87.36–98.95%]	100.00%	100.00%	95.05% [90.82–99.28%]	97.04% [94.49–99.60%]
	VREfm	100.00%	93.15% [87.36–98.95%]	95.05% [90.82–99.28%]	100.00%	97.04% [94.49–99.60%]
Validation Set	VSEfm	90.16% [82.69–97.64%]	100.00%	100.00%	91.67% [85.28–98.05%]	95.28% [91.59–98.97%]
	VREfm	100.00%	90.16% [82.69–97.64%]	91.67% [85.28–98.05%]	100.00%	95.28% [91.59–98.97%]

VSEfm, vancomycin-susceptible *Enterococcus faecium*; VREfm, vancomycin-resistant *Enterococcus faecium*.

### 3.3 Validation of characteristic peaks in the Meizhou area

**Figure 4:**
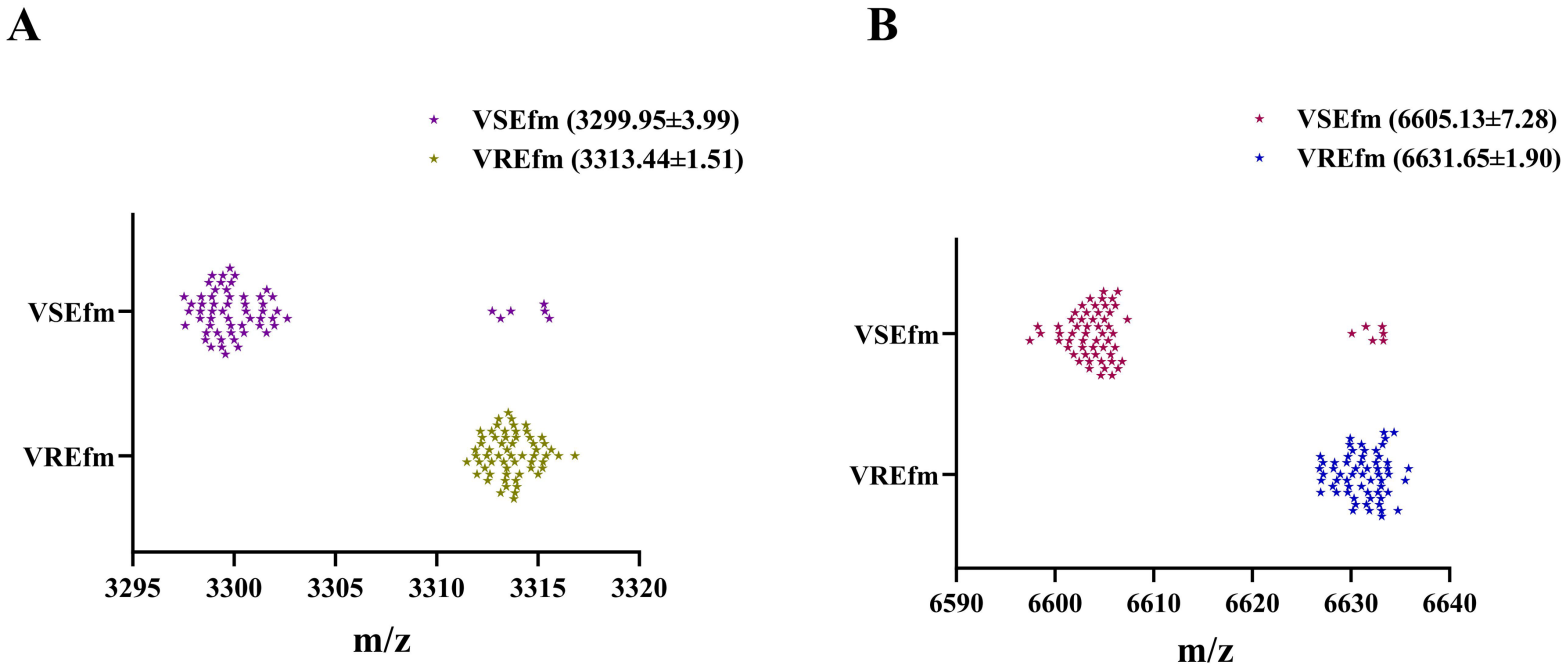
Scatter plots of the characteristic peaks of the VREfm and VSEfm strains from the validation set. The characteristic peaks of 61 VSEfm isolates and 66 VREfm isolates located at m/z 3299.95 ± 3.99 and m/z 3313.44 ± 1.51, respectivley **(A)** and at m/z 6605.13 ± 7.28 and m/z 6631.65 ± 1.90, respectively **(B)**.

To systematically evaluate the differentiation performance of the characteristic peaks in the Meizhou region, this study collected 127 independent *E. faecium* strains as a validation set for methodological validation. Preliminary mass spectrometry analysis of the protein fingerprint spectra identified 55 VSEfm and 72 VREfm strains. After confirmation by the MIC method, the final identification results for the 127 *E. faecium* strains were 61 VSEfm and 66 VREfm. Comparative analysis revealed that the mass spectrometry method misclassified 6 VSEfm strains as VREfm (Figure 4). A 2 × 2 contingency table was constructed based on the identification results of the MIC method and the mass spectrometry method (Table 3), and the following diagnostic performance metrics were calculated: sensitivity 100.00%, specificity 90.16% (95% CI: 82.69–97.64%), positive predictive value 91.67% (95% CI: 85.28–98.05%), negative predictive value 100.00%, and overall agreement rate 95.28% (95% CI: 91.59–98.97%) (Table 2). The validation results were highly consistent with the training set analysis, confirming the reliability of the method. Based on this, we established a MALDI-TOF MS rapid identification criterion: when an *E. faecium* strain exhibits both characteristic peaks at m/z 3313.44 ± 1.51 and m/z 6631.65 ± 1.90, it is classified as VREfm; if both characteristic peaks at m/z 3299.95 ± 3.99 and m/z 6605.13 ± 7.28 are present, it is classified as VSEfm.

**Table 3:** 2 × 2 contingency table for the validation set based on identification results by MIC and mass spectrometry method.

Identification method		MIC		Total (Number of Strains)
		VREfm (Number of Strains)	VSEfm (Number of Strains)	
MALDI-TOF MS	VREfm (Number of Strains)	66	6	72
	VSEfm (Number of Strains)	0	55	55
Total (Number of Strains)		66	61	127

AST, antimicrobial susceptibility testing; MIC, minimum inhibitory concentration; MALDI-TOF MS, matrix-assisted laser desorption ionization time-of-flight mass spectrometry; VSEfm, vancomycin-susceptible *Enterococcus faecium*; VREfm, vancomycin-resistant *Enterococcus faecium*.

## 4 Discussion

In recent years, researchers have developed various rapid methods based on mass spectrometry technology to distinguish between antibiotic-sensitive and resistant strains. These methods primarily rely on key differential features such as the absence or presence of characteristic peaks, significant changes in peak signal intensity, and spatial shifts in peak coordinates. However, these studies also have shown that the characteristic peaks exhibit notable regional variability. For instance, in the case of carbapenem-resistant *Klebsiella pneumoniae*, the ST258 strain prevalent in Europe and America is characterized by a peak at m/z 11,109 (Gaibani et al., 2016; Youn et al., 2016), whereas the ST11 strain dominant in China is identified by a peak at m/z 4,521 (Song, 2020; Zeng et al., 2022). Regarding VREfm research, Griffin et al. (2012) were the first to identify a stable characteristic peak at m/z 5094.7 in *vanB*-type VREfm, which was later confirmed to be encoded by the *hirJM79* gene as a hiracin secretion protein (Brackmann et al., 2020). However, this characteristic peak at m/z 5094.7 is not applicable to the *vanA*-type strains predominant in Asia (Figures 1A,B). To the best of our knowledge, no stable common peak has been identified for *vanA*-type strains to date. Notably, even among the *vanA*-type VREfm strains circulating in different provinces of China, significant differences in the identified resistance peaks have been observed (Figure 1B). Our results herein showed that all the tested VREfm strains isolated in Meizhou harbor *vanA* gene and displayed two resistance peaks at m/z 3313.44 ± 1.51 and 6631.65 ± 1.90 (Figures 2A,B), however, these two peaks were not reported in the VREfm strains isolated from multiple regions of China (Figure 1B). It is worthy noting that the peak at m/z 6631.65 ± 1.90 aligns closely with the *vanA*-type VREfm peak at m/z 6,630 identified by Griffin et al. (2012). Similarly, the characteristic peaks for VSEfm were m/z 3299.95 ± 3.99 and m/z 6605.13 ± 7.28, distinctly from the previously reported peaks at m/z 3,301–3,304 (Wang et al., 2021; Wang et al., 2022). These results together highlight the distinct regional epidemiological characteristics of the resistance-associated peaks, thereby limiting their broad clinical application. Nevertheless, these characteristic peaks remain effective in distinguishing locally prevalent VSEfm and VREfm strains. Both the training set and the validation set showed that the characteristic peaks identified in this study had sensitivity, specificity, and overall agreement rates far more exceeding 90% (Table 2 and Figures 2C,D, 3, 4) indicating an excellent differentiation performance for VSEfm and VREfm strains prevalent in the Meizhou region, thus providing an applicable and rapid method for clinical management and precise medicine.

The observed differences in characteristic peaks across regions may be attributed to the following factors: (1) genetic diversity and regional adaptation of strains, *E. faecium* strains, widely distributed across different geographical regions, exhibit genetic diversity, leading to region-specific distribution of sequence types. Additionally, regional environmental pressures, such as local antibiotic usage patterns and living habits, may drive the evolution of unique adaptive mechanisms in these strains. These factors collectively contribute to variations in protein expression profiles, which in turn influence the formation of characteristic peaks in MALDI-TOF MS spectra (Wang et al., 2018; Wang et al., 2019). (2) Diversity in resistance mechanisms. VREfm strains acquire and express various resistance genes to, confer vancomycin resistance. The prevalence of these resistance genes varies significantly among VREfm strains in different regions, resulting in diverse resistance mechanisms and the formation of region-specific peaks in mass spectra. (3) Differences in algorithms and models. Variations in data processing algorithms and analytical methods used by different MALDI-TOF MS systems contribute to discrepancies. Previous studies showed that different algorithms, such as ClinProTools, Supervised, and Convolutional Neural Networks, exhibit varying sensitivities and specificities in peak extraction and identification, potentially leading to different characteristic peaks under varying analytical conditions (Wang et al., 2021; Wang et al., 2022). (4) Instrumentation and operational variability. The stability and reproducibility of mass spectra are influenced by multiple experimental factors, including sample purity, the mixing ratio of matrix solution to sample, and crystallization uniformity. Furthermore, differences in key performance parameters such as resolution, sensitivity, and mass accuracy among various mass spectrometer models may cause variations in peak intensity, signal-to-noise ratio, and mass-to-charge (m/z) values, thereby affecting the specificity and stability of characteristic peaks (Yoon and Jeong, 2021; Wolters et al., 2011).

There are several bottlenecks in the current MALDI-TOF MS technology for identifying VREfm. Firstly, the technique lacks standardized operational procedures to distinguish between VREfm and VSEfm, and its sensitivity and specificity have not yet reached the ideal level of 100%. As a result, the identification results can only serve as a clinical reference and cannot fully replace traditional detection methods. Secondly, since characteristic peaks exhibit significant regional epidemiological features, the identification systems established in different areas have geographical limitations. The samples analyzed in this study were solely from the Meizhou region, which may limit the generalizability of our findings to the VSEfm and VREfm strains from other regions. Lastly, with the emergence of new resistance mechanisms, the expression of related regulatory proteins may change, rendering existing characteristic peaks no longer applicable and posing a risk of misidentification.

To address these technical bottlenecks, we propose a systematic optimization strategy. (1) At the experimental level, a standardized sample pretreatment system must be established and optimized by each lab. The bacterial culturing parameters, including incubation time, medium selection, and culture conditions, should strictly set and the protein extraction procedures, including but not limited to formic acid concentration and extraction time, should be optimized to ensure the reproducibility of mass spectrometry data. Simultaneously, rigorous control of instrument calibration and matrix coating quality (e.g., homogeneity of HCCA matrix and spotting size) is essential to minimize background signal interference, thereby improving the reliability of characteristic peak detection. (2) In data analysis, continuously integrating regional epidemic strain spectral data and dynamically updating reference databases can effectively enhance the discrimination of borderline resistance phenotypes. Coupled with machine learning algorithms for in-depth analysis of characteristic spectral patterns of resistance-related biomarkers, detection sensitivity can be further improved (Yang et al., 2025; Wang et al., 2022). During optimization, molecular verification techniques such as PCR serve as auxiliary tools for cross-validating ambiguous results. Furthermore, by increasing strain sample sizes and conducting multicenter validation, in-depth analysis of strain genetic variation characteristics will refine the criteria for characteristic peak determination, ultimately improving the accuracy and clinical utility of VREfm identification. This multidimensional optimization strategy forms a closed-loop quality control system from experimental operations to data analysis, providing a practical solution to enhance the clinical value of MALDI-TOF MS in antimicrobial resistance (AMR) surveillance.

In summary, we identified two pairs of characteristic peaks for distinguishing VSEfm and VREfm using MALDI-TOF MS, which is applicable to most *E. faecium* strains prevalent in the Meizhou region. The application of this MALDI-TOF MS method will effectively enhance the local AMR prevention and control capacity. Our future studies will focus on the determination of the proteins behind these characteristic peaks and further validation of these characteristic peak in more VSEfm and VREfm strains from Meizhou and other regions.

## Data Availability

The original contributions presented in the study are included in the article/supplementary material, further inquiries can be directed to the corresponding author.
